# Amination of Non-Functional Polyvinyl Chloride Polymer Using Polyethyleneimine for Removal of Phosphorus from Aqueous Solution

**DOI:** 10.3390/polym14091645

**Published:** 2022-04-19

**Authors:** Sok Kim, Yun Hwan Park, Yoon-E Choi

**Affiliations:** 1 Division of Environmental Science and Ecological Engineering, Korea University, Seoul 02841, Korea; sokkim81@korea.ac.kr (S.K.); sug4393@korea.ac.kr (Y.H.P.); 2 OJeong Resilience Institute, Korea University, Seoul 02841, Korea

**Keywords:** phosphorus, adsorption, adsorbent, polyvinyl chloride, polyethyleneimine (PEI)

## Abstract

The eutrophication of freshwater environments caused by an excess inflow of phosphorus has become a serious environmental issue because it is a crucial factor for the occurrence of harmful algal blooms (HABs) in essential water resources. The adsorptive removal of phosphorus from discharged phosphorus containing effluents has been recognized as one of the most promising solutions in the prevention of eutrophication. In the present study, a polyvinyl chloride (PVC)-polyethyleneimine (PEI) composite fiber (PEI-PVC) was suggested as a stable and recoverable adsorbent for the removal of phosphorus from aqueous phases. The newly introduced amine groups of the PEI-PVC were confirmed by a comparison between the FT-IR and XPS results of the PVC and PEI-PVC. The phosphorus sorption on the PEI-PVC was pH dependent. At the optimum pH for phosphorus adsorption (pH 5), the maximum adsorption capacity of the PEI-PVC fiber was estimated to be 11.2 times higher (19.66 ± 0.82 mg/g) than that of conventional activated carbon (1.75 ± 0.4 mg/g) using the Langmuir isotherm model. The phosphorus adsorption equilibrium of the PEI-PVC was reached within 30 min at pH 5. From the phosphorus-loaded PEI-PVC, 97.4% of the adsorbed amount of phosphorus on the PEI-PVC could be recovered by employing a desorption process using 1M HCl solution without sorbent destruction. The regenerated PEI-PVC through the desorption process maintained a phosphorus sorption capacity almost equal to that of the first use. In addition, consistently with the PVC fiber, the PEI-PVC fiber did not elute any toxic chlorines into the solution during light irradiation. Based on these results, the PEI-PVC fiber can be suggested as a feasible and stable adsorbent for phosphorus removal.

## 1. Introduction

Phosphorus (P) is widely applied as an important element in various areas of human activity (e.g., agricultural, living, and bio-industrial areas) [1]. Accordingly, a large number of effluents possessing phosphorus have been discharged into water bodies (e.g., rivers and lakes) from various point and non-point routes, such as mining, industrial, sewage, agricultural soil, and surface runoffs [2,3,4]. Excessively accumulated phosphorus in water bodies can cause eutrophication, which, in turn, causes serious environmental problems such as harmful algal blooms (HABs) in precious water resources because phosphorus is a limiting nutrient for the proliferation of harmful microalgal and cyanobacterial cells [5]. Therefore, the control and removal of phosphorus from aqueous phases is an immensely important task to maintain the quality of water resources.

Various water treatments based on physiochemical and biological technologies, such as electrodialysis, struvite precipitation, membrane filtration, and biological digestion, are being developed and applied to remove phosphorus from aqueous phases [6,7,8,9,10]. Among these methods, chemical precipitation is used to form ammonium phosphate, is recognized as the most well-established phosphorus removal process, and is reported to have the highest removal efficiency (90–95%); however, it can be economically applied to the treatment of an aqueous solution containing a high phosphate concentration (>50 mg/L). Moreover, in the case of biological and membrane processes, some technical limitations have been claimed, such as low effectiveness and strict control requirements (biological process) and high capital and energy cost requirements (membrane process).

Adsorption technology is considered as a reliable and economical application method for the removal of phosphorus from aqueous solutions, because it does not require any additional separate operation processes, additives, or power sources [11]. Therefore, various types of adsorbents have been designed for application in the adsorption process for the removal of phosphorus (phosphate) from aqueous phases. Synthesized metal hydroxide/oxide-based adsorbents, such as Mg(OH)_2_ and ZrO_2_, have been reported to have considerable phosphate adsorption capacities at 16.3 and 47.4 mg/g, respectively [12]. Clay minerals, such as palygorskite [13] and clinoptilolite [14], have been investigated as adsorbents for the removal of phosphate from aqueous phases. In addition, biochars derived from wood, corncob, rice husk, and saw dust have gained interested as possible adsorbents for the adsorptive removal of phosphate [15]. However, it is difficult to recover these adsorbents from solution after application due to their small size. When considering phosphorus industrial demands and exhausting natural phosphorus sources, and since phosphorus-loaded adsorbents can be another source of phosphorus, the recovery of adsorbent after use may be important from the viewpoint of industrial sustainability [3]. Molded adsorbents using a polymer matrix have been suggested as the solution for the difficult separation of small, formed adsorbents from solution. For example, chitosan is recognized as a potent polymer matrix of adsorbent for the removal of anionic-formed phosphates by electrostatic attraction due to its content of numerous cationic amine groups and possibility to be immobilized in various hydrogel forms (bead and fibers) [16]. However, since it is a biodegradable biopolymer, it is possible to re-discharge adsorbed phosphorus from chitosan-based adsorbents through natural decomposition of the matrix. Indeed, it has been reported that adsorbed cyanotoxins on chitosan can be released again into the aqueous phase through the degradation of the chitosan matrix [17]. Therefore, to prevent secondary pollution by re-discharging target pollutants, the use of a stable matrix should be considered in the development of molded adsorbents.

In the present study, to fabricate an easily recoverable and highly stable adsorbent for the adsorptive removal of phosphorus, the synthetic polymer polyvinyl chloride (PVC), which is a chemically/physically stable, inexpensive, and light-weight commercial thermoplastic [18], was applied as an adsorbent matrix. Since the adsorption target phosphorus generally appears in anionic phosphate forms (H_2_PO_4_^−^, HPO_4_^2−^, and PO_4_^3−^) in aqueous phases depending on its pK_a_ property, it can be electrostatically adsorbed to cationic functional groups such as amine groups. However, the chemical structure of PVC does not possess any functional groups as adsorption sites for anionic phosphates. To aminate the PVC matrix, amine-rich ionic polymer polyethyleneimine (PEI) was applied because PEI modification is a well-known method that can efficiently enhance the adsorption performance of adsorbents for anionic pollutants, including phosphates [2,19,20]. Generally, to fabricate PEI-modified molded adsorbents, at least three separate reaction processes are required, including adsorbent molding, PEI-coating/grafting, washing, and cross-linking processes. However, we expected that the PEI-PVC composite adsorbent (PEI-PVC) would only require polymer dissolution and fabrication processes because the amine groups of PEI molecules can be coupled to the alkyl chloride groups of the PVC backbone via the nucleophilic substitution reaction through chloro-groups during the polymer dissolution step [21]. Consequently, we could fabricate the PEI-PVC adsorbent through the direct injection of the dissolving PEI-PVC composite solution into water. In addition, to obtain an easily separable and surface area-maximized PEI-PVC adsorbent, it was fabricated in fiber form. The enhanced amine groups and decreased chloride of the PEI-PVC fiber compared to those of the pristine PVC could be confirmed using FT-IR and XPS analyses. Through pH edge, kinetics, and reuse tests using the PEI-PVC fiber for phosphorus, the pH-dependent, rapid, and reusable phosphorus adsorption properties of the PEI-PVC fiber were determined. The estimated phosphorus adsorption capacity of the PEI-PVC through isotherm tests was comparable to that of other reported adsorbents. Moreover, during utilization as an adsorbent, although the pristine PVC matrix could generate toxic chlorines by photo-oxidative degradation, the PEI-PVC fiber did not elute chlorines under light irradiation conditions. Therefore, the suggested PEI-PVC fiber can be feasible and stable adsorbent for phosphorus adsorption. In addition, our results might provide a sustainable way to valorize waste PVC to a capable adsorbent for the treatment of anionic pollutants.

## 2. Materials and Methods

### 2.1. Materials

Polyvinyl chloride (PVC, MW of 40 kDa), branched polyethyleneimine (PEI) solution (MW of 750,000, 50% PEI content), and potassium phosphate dibasic (K_2_HPO_4_, >98%) were purchased from Sigma-Aldrich Korea Ltd. (Seoul, Korea). *N*,*N*-dimethylformamide (DMF, 99.8%) was supplied by Duksan Science (Seoul, Korea). HCl (36%) and NaOH (>97%) were purchased from Samchun Chemicals Co., Ltd. (Seoul, Korea) and Daejung Chemicals & Metals Co., Ltd. (Siheung, Korea), respectively.

### 2.2. Preparation of Adsorbents

To prepare the PEI composite PVC fiber sorbent (PEI-PVC), two grams of PEI solution (50% in solution) was mixed with 30 mL of PVC solution (2 g PVC/30 mL DMF). Next, the mixture was allowed to react at 80 °C for 4 h in a water bath. The reacted mixture was continuously injected into a methanol solution (50% *v*/*v* in D.W.) to form a fiber sorbent using a plastic hub needle with air compression. The fabricated PEI-PVC fibers were washed with distilled water several times for the removal of residual DMF and other impurities. The prepared PEI-PVC fiber was then freeze-dried for 24 h. To minimize the effect of humidity and CO_2_ on the sorbent, it was stored in a desiccator during period adsorption tests.

### 2.3. Comparison of Functional Group Characteristics in PVC and PEI-PVC Fibers

The measurement of functional group characteristics on the sorbents was carried out using Fourier transform infrared spectroscopy (FT-IR, Agilent Cary 630 FTIR, Agilent Technology, Santa Clara, CA, USA). FT-IR analyses of adsorbents were conducted within the wavenumber range of 650–4000 cm^–1^ in the attenuated total reflectance (ATR) mode with scanning number of 100 and 0.9 cm^−1^ scanning resolution. Furthermore, to analyze minute changes in the functional groups of the sorbents, the XPS signals for N_1s_ and Cl_2p_ of the PVC and PEI-PVC were analyzed via XPS (X-TOOL, ULVAC-PHI, Kanagawa, Japan).

### 2.4. Evaluation of Phosphorus Adsorption Performance of Adsorbents

To determine and compare the phosphorus adsorption performances of the PEI-PVC and AC, pH effect (pH edge), kinetics, and isotherm tests were performed in a batch system. In the adsorption experiments, the phosphorus stock solution (initial concentration of phosphorus: 1000 mg/g) was diluted with distilled water to prepare test solutions containing desired initial concentration of phosphorus. For the adsorption test, the adsorbents (adsorbent dosage: 1 g/L) were agitated with the prepared phosphorus solutions in a shaking incubator until reaching the adsorption equilibrium state at 170 rpm and 25 °C. In addition, during the adsorption process, the solution pH was continuously monitored and adjusted to the desired pH range using HCl (0.1 M and 1 M) and NaOH (0.1 M and 1 M) solutions. The detailed conditions of each experiment are summarized in Table 1.

After adsorption processes, to measure residual phosphorus concentration in the experimental samples, liquid–solid separation was conducted using high-speed centrifugation at 9000 rpm. The residual phosphorus concentration in the supernatants was detected using inductively coupled plasma-optical emission spectrometry (ICP-OES, Agilent, Santa Clara, CA, USA). The adsorbed amount of phosphorus by the adsorbents could be calculated using Equation (1) as presented below:

(1) $$q=\frac{V_{i}C_{i}-V_{f}C_{f}}{M}$$

where *q* (mg/g) is the phosphorus adsorption capacity of the adsorbent. *M* (g) is the applied adsorbent weight to the adsorption experiment. *C_i_* and *C_f_* are the phosphorus concentrations (mg/L) at the initial and final adsorption states, respectively. *V_i_* (L) is the initial volume of the sample, and *V_f_* (L) is the final volume considering the amount of added acid/alkaline solution for pH adjustment.

### 2.5. Determination of PEI-PVC Reusability

To determine adsorbent reusability of the used PEI-PVC fiber in phosphorus adsorption, phosphorus-loaded PEI-PVC fiber was prepared through adsorption process (agitating 0.03 g of PEI-PVC fiber with 30 mL of phosphorus solution for 4 h at pH 5 and 25 °C). The initial phosphorus concentration was 98.25 ± 0.30 mg/L. After the sorption process, phosphorus-loaded PEI-PVC was separated from the mixture and briefly rinsed using 30 mL of distilled water one time to remove residual phosphorus solution. Then, the rinsed phosphorus-loaded PEI-PVC was treated in 30 mL of 1M HCl solution for 4 h to dissociate the adsorbed phosphorus from used PEI-PVC. After that, the regenerated PEI-PVC was washed several times using distilled water to remove the remaining HCl solution. The adsorption process was performed again using completely regenerated PEI-PVC under the same conditions as the first adsorption trial. The adsorption and desorption processes using PEI-PVC were repeated in three cycles.

### 2.6. Measurement of Chlorine Elution from PVC-Based Sorbents

To measure the eluted chlorine levels that were generated from the PVC backbone of the sorbents, PVC and PEI-PVC fibers were agitated in 150 mL of autoclaved distilled water for 24 h under white fluorescent light (50 mol/m^2^) at 25 °C. The eluted chlorine concentration in the gathered samples was measured using a residual chlorine meter (HI 96710, Hanna, Woonsocket, RI, USA) following the manufacturer’s protocol. All experiments were conducted thrice.

## 3. Results and Discussion

### 3.1. Change of Functional Group Properties of PVC Sorbent after PEI Reaction

**Figure 1:**
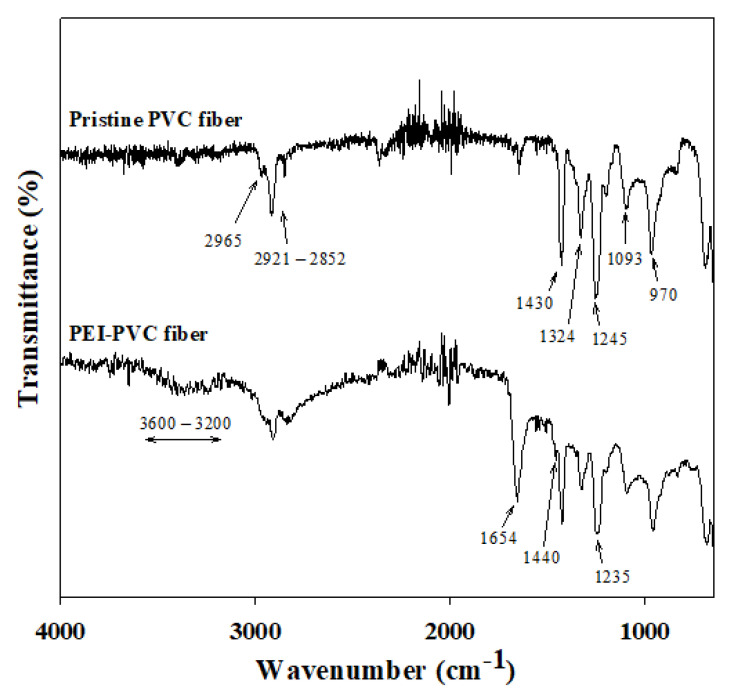
FT-IR spectrum of PVC and PEI-PVC.

**Figure 2:**
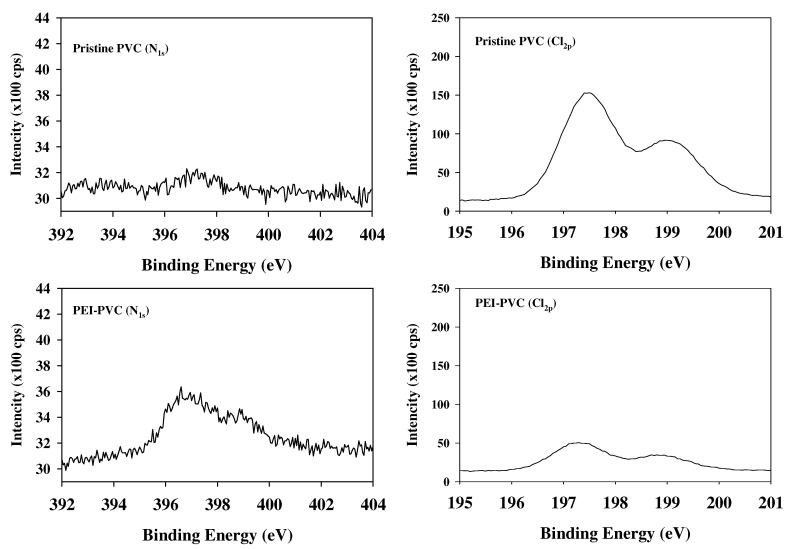
XPS N1s and Cl2p signals of PVC-based sorbent materials.

Since the adsorption target phosphorus usually exhibits anionic phosphate forms in an aqueous solution depending on its pK_a_ properties, cationic functional groups possessing sorbents can adsorb phosphates by electrostatic attraction. Therefore, the determination of the existing cationic binding sites on the sorbents might be an important factor to confirm the applicability of adsorbents to phosphorus adsorption processes. In the present study, the newly generated cationic functional groups of the PEI-PVC fiber by the PEI and PVC composite were analyzed using FT-IR and XPS instruments. Figure 1 presents the FT-IR spectrum of the PVC and PEI-PVC. As shown in Figure 1, the PEI-PVC fiber showed FT-IR peaks associated with the base material PVC. The peaks in the range of 2921–2852 cm^−1^ correspond to the symmetric and symmetrical stretching bonds of C–H in the PVC molecules [22,23]. It has been reported that peaks at 2965 and 1245 cm^−1^ are related to the C–H stretching of CH–Cl in the PVC molecule [24]. In addition, the peaks at 1430 (C–H bending of –CH_2_), 1324 (C–H deforming in plane), 1093 (C–C stretching), and 970 cm^−1^ (CH_2_ rocking) indicate the chemical bond properties of the PVC matrix [25]. In the FT-IR spectra of the PEI-PVC, FT-IR adsorption peaks associated with amine groups could be found, whereas those of the PVC did not show. The newly observed broad peak in the range of 3600–3200 cm^−1^ and the strong peak at 1654 cm^−1^ are attributed to the asymmetric N–H stretching of amines and the bending of secondary amines, respectively [26]. In addition, the appearance of the small peak at 1440 cm^−1^ can be attributed to –NH bending or C–N stretching [26,27]. Furthermore, the intensity of the peak at 1245 cm^−1^ assigned to the bending bond of C–H near Cl in the PVC backbone decreased and shifted to 1235 cm^−1^ in the FT-IR spectra of the PEI-PVC. These observed peak changes in the FT-IR result of the PEI-PVC fiber might indicate increased amine groups and decreased Cl amounts in the PEI-PVC fiber due to a nucleophilic substitute reaction between the chloro-groups of the PVC backbone and the amine groups of the PEI molecules [21]. Additional evidence related to these changes in functional groups could be confirmed by XPS analyses for nitrogen (XPS N_1s_) and chloride (XPS Cl_2p_) atoms of the PVC-based fibers (Figure 2). When comparing the XPS N_1s_ signals between the PVC and PEI-PVC fibers, the PVC fiber did not show any XPS signals related to the nitrogen-based chemical bonds (i.e., amine groups). However, in the case of the PEI-PVC, the strong N_1s_ signal was newly observed in the binding energy range of 392–404 eV. This signal might be attributed to the amine groups of the coupled PEI molecules with the PVC backbone since N_1s_ peaks of –NH_2_ and C–N = C bonds in the PEI molecules could be observed at 398.8 and 399.5 eV [19]. This result is clearly connected to the newly appeared FT-IR peaks of the amine groups in the FT-IR spectra of the PEI-PVC fiber. In the case of XPS Cl_2p_ signals, a higher XPS signal intensity was observed in the XPS result of the PVC than in that of the PEI-PVC. This evidently indicates that the amount of chloride was decreased in the PEI-PVC fiber compared to that of the PVC fiber owing to the substitution reaction between the PEI molecules and the chloride in the PVC.

**Figure 3:**
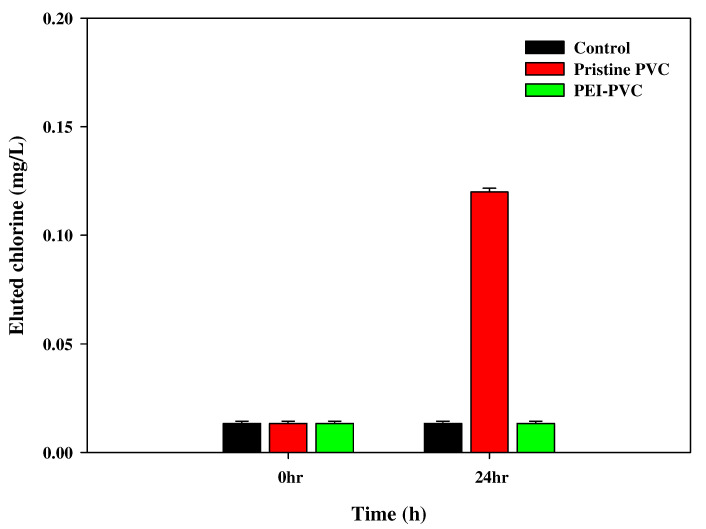
Eluted chlorine levels from PVC and PEI-PVC in an aqueous solution.

The decreased amount of chloride in the PEI-PVC compared to the pristine PVC could be additionally supported by the different eluted chlorine concentrations from the PVC and PEI-PVC fibers under light irradiation conditions. It has been reported that PVC can generate toxic chlorines via the photo-degradation of the backbone structure [28]. As shown in the XPS Cl_2p_ results, since the amount of chloride in the PEI-PVC was reduced, it was expected that the elution of chlorines from the PEI-PVC fiber might be reduced compared to that of the PVC. Therefore, the generated chlorines from the PVC and PEI-PVC fibers were compared (Figure 3). As we hypothesized, the pristine PVC fiber released 0.12 ± 0.002 mg/L of chlorine into the aqueous solution during light exposure for 24 h, whereas the PEI-PVC fiber did not. The PEI-PVC-agitated sample showed a chlorine concentration almost the same as that of the control water (0.013 ± 0.001 mg/L). Furthermore, the prevention of toxic chlorine emissions from the PEI-PVC fiber under light conditions might be a meaningful result indicating that the PEI-PVC fiber is directly applicable and a stable adsorbent for the removal of phosphorus in actual environments.

### 3.2. pH Effect on Phosphorus Adsorption

Through the FT-IR and XPS measurements, it was determined that amine groups derived from PEI molecules are the main binding sites of the PEI-PVC fiber, which can electrostatically adsorb phosphates. Since the electrostatic attraction between the PEI-PVC fiber and phosphates can be affected by the solution pH due to the deprotonation of the amine groups of the PEI-PVC and the phosphates in the solution depending on their pK_a_ properties, the solution pH condition is an important factor in the adsorption process [2]. In the present study, the pH effect on the phosphorus adsorption of the PEI-PVC was evaluated under different solution pH conditions (pH 2–8). In addition, the pH effect on the phosphorus sorption capacity of the conventional adsorbent activated charcoal (AC) was evaluated to make a comparison with that of the PEI-PVC fiber.

**Figure 4:**
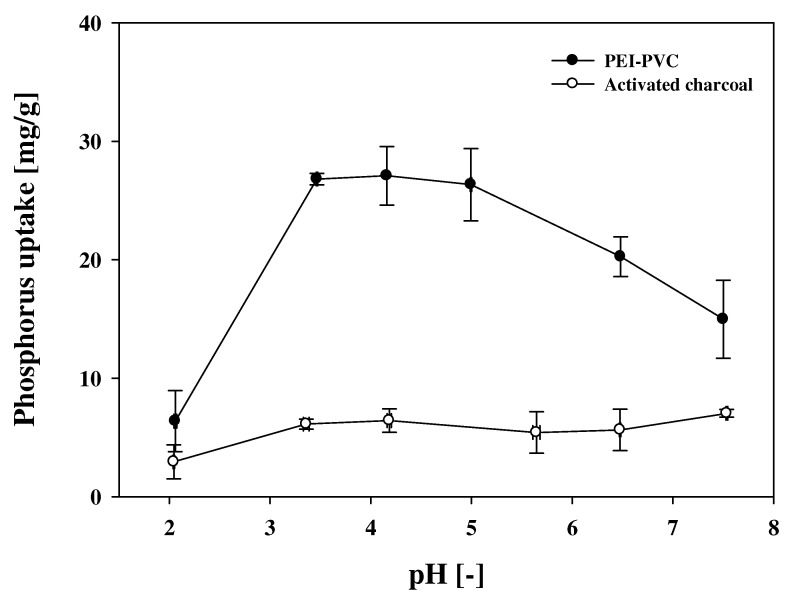
pH effect on the phosphorus sorption uptake of PEI-PVC and activated charcoal.

Figure 4 illustrates the pH effect on the phosphorus uptakes of the PEI-PVC and AC. As displayed in Figure 4, the PEI-PVC exhibited a pH-dependent phosphorus adsorption property. With an increase in solution pH from 2 to 3.5, the phosphorus sorption capacity of the PEI-PVC increased accordingly. At solution pH 3.5, the phosphorus sorption capacity of the PEI-PVC was recorded as approximately 27 mg/g (26.8 ± 0.48 mg/g), and it showed similar phosphorus sorption capacities until pH 5. Above pH 5, the phosphorus uptake of the PEI-PVC fiber decreased substantially. The pH-dependent phosphorus adsorption of the PEI-PVC fiber can be explained by the electrostatic interaction between the amine groups of the PEI-PVC and phosphate anions, as we assumed previously. It has been reported that the pK_a_ values of amine groups (e.g., primary, secondary, and tertiary amine groups) in PEI molecules can be observed at approximately pH 4.5, 6.7, and 11.6, respectively [29]. Accordingly, the amine groups on the PEI-PVC derived from PEI molecules can be fully positively charged under acidic conditions below pH 4; therefore, it was expected that the phosphorus sorption uptake of the PEI-PVC might record the maximum value at pH 2. However, the phosphorus sorption uptake of the PEI-PVC at pH 2 was significantly lower than that at pH 3.5. This result might be attributed to the pH dependence of phosphate speciation. Phosphorus can exist as uncharged phosphoric acid (H_3_PO_4_) and electrostatically adsorbable phosphate anions (H_2_PO_4_^−^, HPO_4_^2−^, and PO_4_^3−^) depending on the solution pH condition in the aqueous solution [30]. According to our previous study [2], only 40% of total phosphorus amounts can be presented as the electrostatically adsorbable phosphate anion H_2_PO_4_^−^ at pH 2. However, the almost amount of phosphorus can be changed to the phosphate anionic form at pH 3.5. The increasing fraction of anionic phosphate might contribute to the increasing phosphorus uptake of the PEI-PVC in the range of pH 2–pH 3.5 by the enhancement of the electrostatic attraction between the PEI-PVC and phosphate. Above pH 5, although the almost amount of phosphorus can be formed as the adsorbable phosphate anions, since the positivity of the PEI-PVC could be lost by the deprotonation of amine groups depending on their pK_a_ properties, the phosphorus sorption capacity of the PEI-PVC might be reduced. The conventional adsorbent, AC, did not show pH dependence on phosphorus adsorption capacity. However, the phosphorus sorption capacities of the AC were significantly lower than those of the PEI-PVC fiber under all of the pH conditions.

### 3.3. Reusability of PEI-PVC Fiber

**Figure 5:**
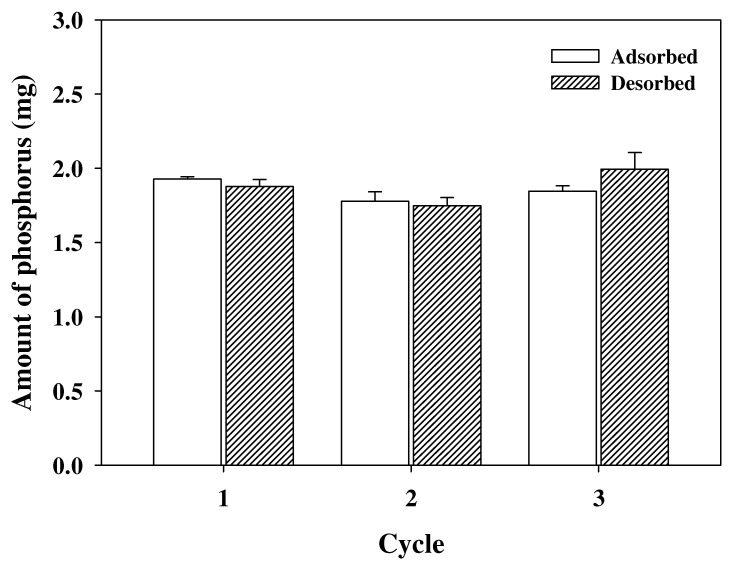
Reusability of PEI-PVC in three cycle applications (adsorbed phosphorus at pH 5 and desorbed in 1 M HCl solution).

The pH-dependence property of the PEI-PVC fiber on phosphorus adsorption could be applied to the phosphorus desorption process for the reuse of the PEI-PVC. As discussed above, under highly acidic conditions, since phosphate anions can be changed to uncharged phosphoric acid (H_3_PO_4_), adsorbed phosphorus might be recovered from phosphate-loaded PEI-PVC. To estimate the desorbed amount of phosphorus, the phosphate-adsorbed PEI-PVC at pH 5 was treated using 1M HCl solution. Figure 5 displays the measured amounts of the adsorbed and desorbed phosphorus through the sorption and desorption processes using the PEI-PVC fiber. As shown in Figure 5, 1.93 ± 0.01 mg of phosphorus could be adsorbed on the PEI-PVC at pH 5 with the first use of the PEI-PVC fiber. In addition, after the treatment of the phosphate-loaded PEI-PVC using the 1M HCl solution, the almost amount of adsorbed phosphorus on the PEI-PVC fiber could be desorbed (1.88 ± 0.05 mg) owing to the loss of electrostatic attraction between the binding site and the sorbate through the speciation change from phosphate anions to uncharged phosphoric acid under highly acidic conditions. From the replicated adsorption and desorption tests, it was found that the regenerated PEI-PVC could adsorb a similar amount of phosphorus to that used in the first trial. In the second and third trials of phosphorus adsorption using regenerated PEI-PVC, 1.78 ± 0.06 and 1.84 ± 0.04 mg of phosphorus were adsorbed on the regenerated PEI-PVC, respectively. When considering the reusability of PEI-PVC, the adsorptive removal process of phosphorus using PEI-PVC might have an economic benefit.

### 3.4. Adsorption Kinetics

**Figure 6:**
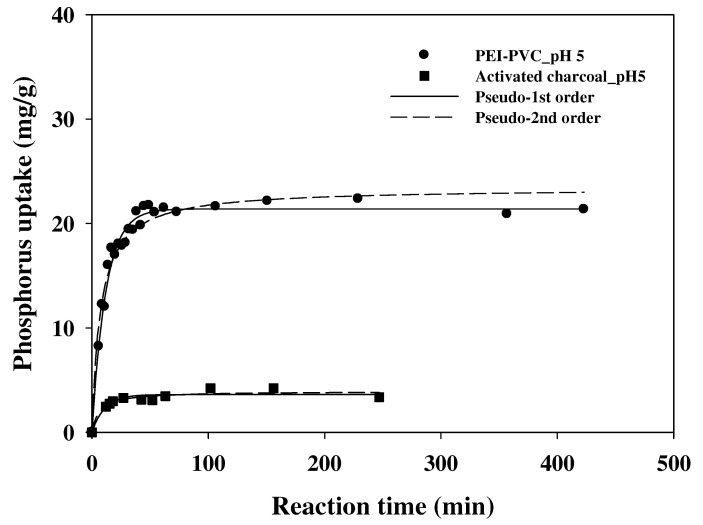
Phosphorus sorption kinetics of PEI-PVC and activated charcoal at pH 5.

Adsorption equilibrium time is an important factor to determine the efficiency of the developed sorbent for the target materials. Therefore, to determine the sorption equilibrium time of the PEI-PVC for phosphorus, a kinetic experiment using the PEI-PVC was performed in a batch system at pH 5. From the kinetic result (Figure 6), the PEI-PVC showed a rapid adsorption equilibrium at approximately 30 min for phosphate anions at pH 5. In the case of the AC, a faster phosphorus adsorption equilibrium was attained than that attained with the PEI-PVC (within 15 min). However, this might be attributable to the significantly low phosphorus sorption capacity of the AC compared to that of the PEI-PVC. To compare the phosphorus sorption kinetic parameters of the PEI-PVC and AC in detail, a pseudo-second-order kinetic model was applied to the experimental kinetic data of the PEI-PVC fiber and AC because it is one of the representative kinetic models used to determine the kinetic properties of adsorbents. The pseudo-second-order kinetic model is represented as Equation (2):

(2) $$q_{t}=\frac{q_{e}^{2}k_{2}t}{1+q_{e}k_{2}t}$$

where *q_e_* and *q_t_* are the phosphorus sorption capacity of the sorbents (mg/g) at the sorption equilibrium state and specific sorption reaction time (*t*), respectively. The parameter *k*_2_ (g/mg min) indicates the estimated adsorption rate constants from the pseudo-second-order model, respectively.

The estimated kinetic parameters for the PEI-PVC and AC from the pseudo-second-order kinetic model are summarized in Table 2. According to Table 2, the estimated correlation coefficients (R^2^) of the pseudo-second-order model for the phosphorus sorption kinetic data of the PEI-PVC and AC were 0.9585 and 0.9211, respectively. These high R^2^ values indicate that the phosphorus sorption kinetic experimental results of the PEI-PVC and AC were predicted well by the pseudo-second-order kinetic model. The sorption reaction rate (k_2_) of the AC was estimated as 0.037 ± 0.013 g/mg·min using the kinetic model. This was a value approximately 6.5 times higher than that of the PEI-PVC (0.0057 ± 0.0007 g/mg·min), and it was better connected to the faster sorption equilibrium state of the AC than the PEI-PVC fiber. However, as shown in the experimental kinetic results, the PEI-PVC showed a comparable sorption equilibrium time to that of the AC. Furthermore, the phosphorus sorption capacity (q_e_) calculated from the sorption kinetic data of the PEI-PVC was 5.9 times higher (23.39 ± 0.47 mg/g) than that of the AC (3.94 ± 0.21 mg/g) using the pseudo-second-order model. Based on the kinetics result, the PEI-PVC fiber can be suggested as an efficient substitute for conventional adsorbents (i.e., activated carbons) to rapidly counteract the inflow of phosphorus from the effluents of point or non-point pollutant sources.

### 3.5. Maximum Phosphorus Sorption Capacity of PEI-PVC Fiber

**Figure 7:**
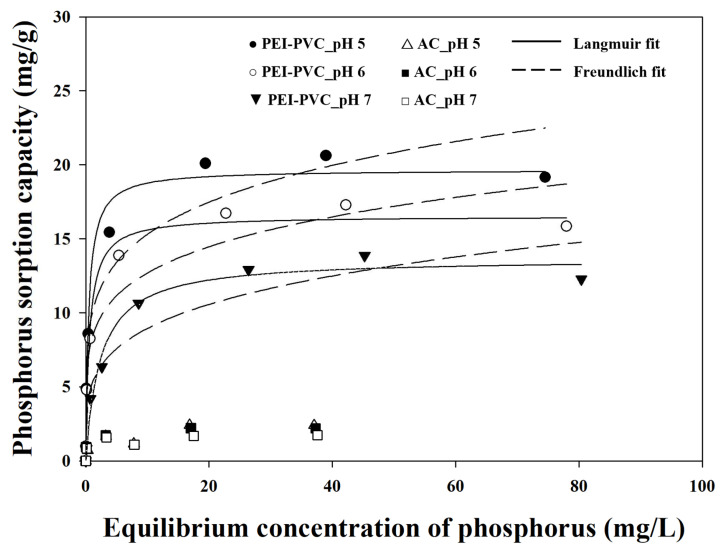
Isotherm results of PEI-PVC and activated charcoal for phosphorus adsorption at pH 5, 6, and 7.

Together with the sorption kinetic property, the maximum sorption capacity (q_max_) of the sorbent toward the target pollutant is an essential factor for the evaluation of the adsorption performance of the developed adsorbent. Therefore, to determine the q_max_ of the developed sorbent PEI-PVC fiber, isotherm experiments were performed under various pH conditions (pH 5–7). In addition, the maximum phosphorus (i.e., phosphate anions) sorption capacities of the PEI-PVC fiber were compared with those of the conventional adsorbent (AC). The obtained isotherm results using the PEI-PVC and AC at different pH values are presented in Figure 7. As shown in Figure 7, the phosphorus sorption capacities of the PEI-PVC and AC showed increasing values as the equilibrium phosphorus concentration was increased. In addition, above the specific equilibrium phosphorus concentration, the phosphorus adsorption uptakes of the PEI-PVC and AC reached the adsorption equilibrium state at all pH values. The phosphorus adsorption uptakes of the PEI-PVC fiber at the equilibrium state decreased from approximately 20 mg/g to 13 mg/g as the solution pH was increased from pH 5 to pH 7. The AC showed almost constant phosphorus adsorption capacities (average 1.75 ± 0.4 mg/g) at the adsorption equilibrium state regardless of pH conditions. These phosphorus uptakes of the AC were clearly low compared to those of the PEI-PVC fiber. Therefore, extensively used isotherm models (Freundlich and Langmuir isotherm model equations) were applied to the only experimental isotherm results of the PEI-PVC for the estimation of detailed isotherm parameters. The Freundlich and Langmuir isotherm model equations are presented as Equations (3) and (4), respectively. The estimated isotherm parameters from these model equations are presented in Table 3.

(3) $$q_{e}=K_{f}C_{f}^{\frac{1}{n}}$$

(4) $$q_{e}=\frac{q_{max,\;L}bC_{f}}{1+bC_{f}}$$

where *q_e_* is the experimentally calculated phosphorus sorption capacity (mg/g). *C_f_* (mg/L) is the final phosphorus concentration in the solution after the adsorption process. The parameters *K_f_* (L/g) and *n* are the Freundlich constants, which can be indicators of sorption capacity and sorption intensity, respectively. The parameters in the Langmuir model equation *q_max,L_* and *b* indicate the maximum capacity (mg/g) and sorption affinity (L/mg) of the adsorbent for phosphorus, respectively.

As summarized in Table 3, the Freundlich parameter (*K_f_*) associated with the phosphorus adsorption capacity of the PEI-PVC fiber increased with the decrease in the solution pH from pH 7 (5.12 ± 0.97 L/g) to pH 5 (9.83 ± 1.34 L/g). The dimensionless n value of the PEI-PVC in phosphorus adsorption showed a similar tendency to that of the *K_f_* values. The recorded n values from the isotherm data of the PEI-PVC fiber for phosphorus at pH 5, pH 6, and pH 7 were 5.21 ± 1.05, 5.29 ± 1.10, and 4.14 ± 0.91, respectively. According to Toor and Jin [31], if the n value is larger than 1, it can be determined that the adsorption process is favorable. Therefore, the phosphorus adsorption process with application of the PEI-PVC fiber might be a favorable process. The application of the Langmuir isotherm model to the experimental isotherm data of the PEI-PVC showed better fitting lines, because of its higher determination coefficients (R^2^ values: 0.9755, 0.9843, and 0.9844 at pH 5, 6, and 7, respectively), than those of the Freundlich model (R^2^ values: 0.9192, 9161, and 0.9046 at pH 5, 6, and 7, respectively) at all solution pH values. The maximum phosphorus sorption capacities (*q_max,L_*) of the PEI-PVC fiber were estimated from the Langmuir model as 19.66 ± 0.82, 16.54 ± 0.54, and 13.66 ± 0.51 mg/g at pH 5, 6, and 7, respectively. In addition, the isotherm result of the PEI-PVC fiber showed a higher *b* value at pH 5 (2.08 ± 0.59 L/mg) than at pH 6 (1.65 ± 0.35 L/mg) and pH 7 (0.42 ± 0.08 L/mg) using the Langmuir model. As discussed above section, since the higher amount of amine groups in the PEI-PVC fiber could be positively activated at pH 5 compared to those at pH 6 and pH 7 depending on the pK_a_ properties of the amine groups, the higher values of the Freundlich and Langmuir isotherm parameters corresponding to the sorption capacity and affinity for phosphorus adsorption could be calculated from the isotherm data.

Furthermore, a comparison of the maximum phosphorus adsorption capacity of the PEI-PVC fiber with that of previously reported adsorbents was performed (Table 4). As shown in Table 4, in general, the adsorbents containing Zr showed higher *q_max_* values than those of the PEI-PVC fiber at natural pH. For instance, the q_max_ values for the phosphorus of Zr-loaded orange waste gel, Zr-loaded MUROMAC, and Zr-loaded okara were calculated as 57, 43, and 14.39 mg/g, respectively. Under the condition of pH 3, the covalently cross-linked chitosan by epichlorohydrin showed higher phosphorus adsorption performance (38.22 mg/g) compared to the PEI-PVC fiber (approximately 27 mg/g), whereas the maximum phosphorus uptake of electrostatically cross-linked chitosan by sodium citrate (13.3 mg/g) was smaller than that of the PEI-PVC. Although we could find several adsorbents showing higher q_max_ values for phosphorus than the PEI-PVC fiber, there still remains an opportunity to enhance the phosphorus adsorption performance of the PEI-PVC fiber through the optimization of adsorbent processes and the additional application of adsorption-supporting materials. In addition, as shown in Table 4, the PEI-PVC fiber still displayed superior or comparable phosphorus adsorption performance to other previously reported sorbents. For example, zirconium ferrite [32], Fe-loaded juniper fiber [33], iron–hydroxided eggshell [34], and La(III)-modified fine needle [35] showed maximum phosphorus sorption capacities of 13, 2.31, 14.49, and 6.31 mg/g at pH 7, respectively.

## 4. Conclusions

In the present study, the amination of a non-functional PVC matrix was performed using a simple chemical reaction process between PEI and PVC to develop an adsorbent for phosphorus removal from aqueous phases. From the FT-IR and XPS-N_1s_ results of the prepared PEI-PVC, the aminated characteristics of the PEI-PVC fiber were well determined. The adsorption of phosphorus on the PEI-PVC was affected by the solution pH. The maximum phosphorus sorption capacity (q_max_) of the PEI-PVC fiber was estimated as 19.66 ± 0.82 mg/g at pH 5 using the Langmuir equation. At pH 6 and pH 7, the q_max_ values of the PEI-PVC fiber were calculated as 16.54 ± 0.54 and 13.66 ± 0.51, respectively. In the kinetic analysis, the PEI-PVC showed a rapid phosphorus adsorption equilibrium time within 30 min at pH 5. The used PEI-PVC could be regenerated without destructing the adsorbent matrix using the 1M HCl solution by the desorption of the adsorbed phosphorus in the PEI-PVC fiber (desorption rate: >98%). In addition, the regenerated PEI-PVC was reusable for phosphorus adsorption without the loss of sorption capacity. Furthermore, it was found that the PEI-PVC fiber did not discharge toxic chlorines during light irradiation. Based on the results, we can suggest that the stable and reusable PEI-PVC fiber can be a feasible adsorbent for phosphorus removal from aqueous phases. However, to further enhance its phosphorus adsorption performance, additional research on the optimization of adsorbent processes and the additional application of adsorption-supporting materials might be needed.

## Figures and Tables

**Table 1:** Adsorption experimental conditions.

Conditions	Experiments		
	pH Effect	Kinetics *	Isotherm
Working volume (L)	0.03	0.08	0.03
Weight of adsorbents (g)	0.03	0.08	0.03
pH	2–8	5 (4.95–5.05)	5, 6, and 7
Initial phosphorus concentration (mg/L)	98.3	91.7	1–100
Adsorption reaction time (h)	24	8	24

* To determine the adsorption equilibrium time of adsorbents, during the adsorption experiment, solution samples were taken at different reaction times.

**Table 2:** Adsorption kinetic parameters of applied sorbents for phosphorus estimated from pseudo-second-order model at pH 5.

Adsorbents	Kinetic Parameters		
	k_2_ (g/mg·min)	q_e_ (mg/g)	R^2^
PEI-PVC	0.0057 ± 0.0007	23.39 ± 0.47	0.9585
AC	0.037 ± 0.013	3.94 ± 0.21	0.9211

**Table 3:** Estimated isotherm parameters of PEI-PVC for phosphorus from Freundlich and Langmuir models.

Models	Parameters	pH		
		5	6	7
Freundlich	*K_f_*	9.83 ± 1.34	8.20 ± 1.14	5.12 ± 0.97
	*n*	5.21 ± 1.05	5.29 ± 1.10	4.14 ± 0.91
	R^2^	0.9192	0.9161	0.9046
Langmuir	*b*	2.08 ± 0.59	1.65 ± 0.35	0.42 ± 0.08
	*q_max, L_*	19.66 ± 0.82	16.54 ± 0.54	13.66 ± 0.51
	R^2^	0.9755	0.9843	0.9844

**Table 4:** Maximum phosphorus adsorption capacities of previously reported adsorbents.

Adsorbent	Maximum Phosphorus Adsorption Capacity (mg/g)	pH Condition	Ref.
Mg(OH)_2_	5.3	pH 7	[12]
ZrO_2_	21.9	pH 7	[12]
Palygorskite	3.7	pH 7	[13]
Clinoptilolite	6.6	pH 5.3	[14]
Zeolite	8.3	pH 5.3	[14]
Fe-loaded juniper fiber	2.31	pH 6.4	[33]
Iron–hydroxide eggshell	14.49	pH 7	[34]
Zirconium ferrite	13	pH 7	[32]
Zr(IV)-loaded apple peels	20.35	pH 2	[36]
Zr(IV)-loaded orange waste gel	57	pH 7	[32]
Zr(IV)-loaded MUROMAC	43	pH 7	[32]
Zn(II)-activated coir pith carbon	5.1	pH 4	[37]
Cross-linked chitosan by epichlorohydrin	38.22	pH 3	[16]
Cross-linked chitosan by sodium citrate	13.3	pH 3	[16]
La(III)-loaded orange waste	13.94	pH 5–7	[38]
La(III)-modified fine needle	6.31	pH 7.1	[35]
Fe(III)-loaded okara	4.78	pH 3	[39]
Zr-loaded okara	14.39	pH 7	[39]

## Data Availability

The data of manuscript is an original research work and has not been published elsewhere.
